# Bystander memory CD8 T cell proliferation after anti-CD40/IL-2 treatment is independent of CD4 T cells

**DOI:** 10.1186/2051-1426-1-S1-P100

**Published:** 2013-11-07

**Authors:** Steven K Grossenbacher, Arta M Monjazeb, Julia Tietze, Gail D Sckisel, Annie Mirsoian, Hui-Hua Hsiao, Brent Koehn, Danice Wilkins, Bruce R Blazer, William J Murphy

**Affiliations:** 1 Dermatology, University of Califonia, Davis, Sacramento, CA, USA; 2 Radiation Oncology, University of Califonia, Davis, Sacramento, CA, USA; 3 Pediatrics, University of Minnesota, Minneapolis, MN, USA

## 

Systemic cancer immunotherapy combining agonistic anti-CD40 and interleukin 2 results in synergistic anti-tumor effects with marked antigen independent expansion of bystander memory CD8 T cells displaying anti-tumor abilities. Our lab has previously shown that this expansion coincides with a loss of peripheral CD4 T cells due to activation induced cell death. While much research to date has focused on the effects of CD4 T cells on antigen-specific CD8 T cell expansion, little is known regarding the role of CD4 T cells in bystander CD8 T cell expansion. Utilizing models of CD4 knockout mice as well as CD4 depleting antibodies we observed a significant expansion of memory CD8 T cells displaying a CD25-NKG2D+ bystander phenotype following immunotherapy, similar to non-depleted mice. Interestingly, the expanded bystander memory population was enriched from cells of the effector memory phenotype and up regulated Tim-3 and PD-1 in the absence of CD4 T cells. However, they also displayed comparable cytokine production and lytic ability suggesting no functional impairment or exhaustion. While Tim-3 and PD-1 expression have previously been linked to exhaustion, the phenotype described here is consistent with their other known role as acute activation markers on effector/effector memory T cells. These results suggest that CD4 T cells may not be necessary for the expansion and activation of antigen-nonspecific bystander memory CD8 T cells under conditions of strong immune stimulation yet may play a role in regulating the conversion of these bystander cells from a central to effector memory phenotype in secondary lymphoid organs.

